# Application of Machine Learning Algorithms to Predict Central Lymph Node Metastasis in T1-T2, Non-invasive, and Clinically Node Negative Papillary Thyroid Carcinoma

**DOI:** 10.3389/fmed.2021.635771

**Published:** 2021-03-09

**Authors:** Jiang Zhu, Jinxin Zheng, Longfei Li, Rui Huang, Haoyu Ren, Denghui Wang, Zhijun Dai, Xinliang Su

**Affiliations:** ^1^Department of Endocrine and Breast Surgery, The First Affiliated Hospital of Chongqing Medical University, Chongqing, China; ^2^Department of Breast Surgery, The First Affiliated Hospital, College of Medicine, Zhejiang University, Hangzhou, China; ^3^Department of Health Statistics, School of Public Health, Chongqing Medical University, Chongqing, China; ^4^Department of Anesthesiology, The First Affiliated Hospital of Chongqing Medical University, Chongqing, China; ^5^Department of General, Visceral, and Transplant Surgery, Ludwig-Maximilians-University, Munich, Germany

**Keywords:** papillary thyroid carcinoma, central lymph node metastasis, machine learning algorithms, lymph node dissections, prediction model

## Abstract

**Purpose:** While there are no clear indications of whether central lymph node dissection is necessary in patients with T1-T2, non-invasive, clinically uninvolved central neck lymph nodes papillary thyroid carcinoma (PTC), this study seeks to develop and validate models for predicting the risk of central lymph node metastasis (CLNM) in these patients based on machine learning algorithms.

**Methods:** This is a retrospective study comprising 1,271 patients with T1-T2 stage, non-invasive, and clinically node negative (cN0) PTC who underwent surgery at the Department of Endocrine and Breast Surgery of The First Affiliated Hospital of Chongqing Medical University from February 1, 2016, to December 31, 2018. We applied six machine learning (ML) algorithms, including Logistic Regression (LR), Gradient Boosting Machine (GBM), Extreme Gradient Boosting (XGBoost), Random Forest (RF), Decision Tree (DT), and Neural Network (NNET), coupled with preoperative clinical characteristics and intraoperative information to develop prediction models for CLNM. Among all the samples, 70% were randomly selected to train the models while the remaining 30% were used for validation. Indices like the area under the receiver operating characteristic (AUROC), sensitivity, specificity, and accuracy were calculated to test the models' performance.

**Results:** The results showed that ~51.3% (652 out of 1,271) of the patients had pN1 disease. In multivariate logistic regression analyses, gender, tumor size and location, multifocality, age, and Delphian lymph node status were all independent predictors of CLNM. In predicting CLNM, six ML algorithms posted AUROC of 0.70–0.75, with the extreme gradient boosting (XGBoost) model standing out, registering 0.75. Thus, we employed the best-performing ML algorithm model and uploaded the results to a self-made online risk calculator to estimate an individual's probability of CLNM (https://jin63.shinyapps.io/ML_CLNM/).

**Conclusions:** With the incorporation of preoperative and intraoperative risk factors, ML algorithms can achieve acceptable prediction of CLNM with Xgboost model performing the best. Our online risk calculator based on ML algorithm may help determine the optimal extent of initial surgical treatment for patients with T1-T2 stage, non-invasive, and clinically node negative PTC.

## Introduction

Papillary thyroid carcinoma (PTC) is one of the most common type of endocrine malignancies with a favorable prognosis (1, 2). Nevertheless, central lymph node metastasis (CLNM), the first station of metastasis, occurs in 30–90% of patients following their first surgery and is correlated with an increased risk of local recurrence (3, 4).

The clinical community has reached a general consensus that central lymph node dissection (CLND) for therapeutic purposes is appropriate in PTC patients with suspected cervical lymph node metastasis (LNM) (5). By contrast, however, there is a growing controversy over the role of prophylactic central lymph node dissection (pCLND) due to the lack of randomized controlled data (6–8). Generally speaking, pCLND is not recommended for a subset of patients with small (T1 or T2), non-invasive, clinically node-negative (cN0) PTC according to the 2015 American Thyroid Association (ATA) guidelines (9), whereas the Japanese Society of Thyroid Surgery and the Chinese Thyroid Association both strongly recommend routine pCLND for cN0 PTC patients in order to stage disease and prevent recurrence. While an incomplete nodal resection in the first surgery may lead to disease recurrence and a second operation (10), it is also important to avoid unnecessary CLND in view of surgical complications such as hypoparathyroidism and recurrent laryngeal nerve injury. Ideal treatment decision-making should be based upon individual patients rather than “one size fits all” approach recommended by guidelines. This highlights the importance of accurate prediction of CLNM occurrence with a more personalized therapeutical schedule.

Machine learning (ML), as a novel type of artificial intelligence (AI), is starting to be widely applied to health-care data analysis (11, 12). By capitalizing on the robust prediction ability of ML algorithms, it may be possible to develop prediction tools which in some cases outperform traditional statistical modeling, and thus giving better prediction of CLNM status. Unfortunately, no current studies have trained ML algorithms to predict CLNM in this subset of PTC.

Hence, the purpose of this study is to develop ML-based models using preoperative and intraoperative clinicopathological characteristics to predict the likelihood of CLNM for individualized treatment and to obtain the best ML algorithms for online CLNM prediction in PTC.

## Methods

### Study Population

We retrospectively retrieved the data of in-patients who underwent thyroid surgery at the Department of Endocrine and Breast Surgery of the First Affiliated Hospital of Chongqing Medical University from December 2016 to December 2018.

### Data Collection

Criteria for inclusion were to be a PTC patient with a tumor size no larger than 40 mm (T1-T2), a non-invasive tumor, and no evidence for lymph nodes metastases (cN0) based on ultrasound (US) data. Tumor size was classified according to the 8th edition of American Joint Committee on Cancer (AJCC) Staging Standards. Criteria for exclusion were distant metastasis, previous thyroid surgery, or incomplete information. This study was approved by the local institutional ethics committee board. Demographic and clinicopathological characteristics data were collected as follows: gender, age, tumor size, tumor location, chronic lymphocytic thyroiditis (CLT), multifocality, bilaterality, and the presence of LNM.

### Surgical Strategy

**Figure 1:**
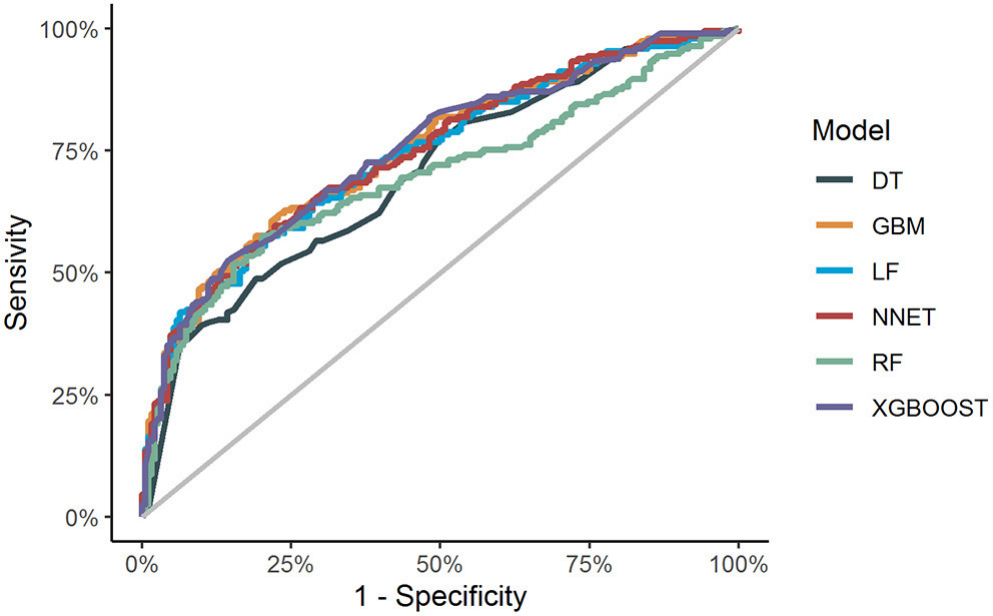
ROC curve analysis of machine learning algorithms for prediction of CLNM patients with T1-T2 stage, non-invasive, and clinically node negative PTC in the validation set. LR, Logistic regression; GBM, Gradient boosting machine; RF, Random forest; DT, Decision tree; NNET, Neural network; Xgboost, Extreme gradient boosting; ROC, receiver operating characteristic; AUC, area under the curve.

At our institution, it is customary to perform pCLND for PTC patients and the detailed surgical procedures were described in previous articles (13, 14). Soft tissues in the prelaryngeal and pretracheal regions were removed and marked as the Delphian (Figure 1) and pretracheal LNs, respectively. Those two subgroups were sent for intraoperative frozen section examination. Then, we proceeded to perform the thyroid lobectomy and ipsilateral paratracheal LN dissection and the paratracheal LN was also sent for frozen section examination. Lastly, all surgical specimens were sent for post-operative histopathologic evaluation. The Delphian lymph node (DLN) was not taken into account in the calculation of central compartment lymph nodes.

### Statistical Analyses

The Fisher's exact test and Student's *t-*test were used for discrete and continuous parameters, respectively. For the independent risk factors of CLNM, a multivariable logistic regression analysis with backward stepwise selection was used to calculate the odds ratios (ORs) with 95% confidence intervals (CIs).

ML algorithm is characterized by its extraordinary performance better than traditional regression approaches in predicting outcomes within large data bases (15–17). In this study, we randomly split our dataset into two groups, namely the training sets (70%) for ML model development and the validation sets (30%) for performance evaluation and we repeated this random splitting until the patient data were equally distributed in both sets (Supplementary Table 1). We developed six types of ML algorithms to model our data: Logistic regression (LR), Gradient boosting machine (GBM), Extreme gradient boosting (XGBoost), Random forest (RF), Decision tree (DT), and Neural network (NNET). In the training process, tuning was considered for ML-based models to avoid overfitting and the best hyper-parameter for ML models was 5-fold cross-validation. Then the ML algorithms were further trained by using the R software to predict the risk of CLNM and we evaluated the predictive ability of each ML classifier, with the same hyper-parameter, in validation sets where the area under the receiver operating characteristic (AUROC) value, and the corresponding sensitivity, specificity, as well as overall accuracy of ML algorithms were all calculated. In the comparison of ML algorithms' performance, the closer to 1 the AUC was, the better the classification model performed. Afterwards, based on the best-performing model, we created an online risk calculator that can make predictions with newly entered PTC patient data, and thus making the risk of CLNM in those patients easily accessible to clinicians. A total of 100 independent training simulation results were used to evaluate the variable importance of each CLNM-predicting ML model. All statistical analyses were performed by using R software, version 3.4.1 (R Foundation for Statistical Computing, Vienna, Austria). The R packages “caret,” “e1071,” “random-forest,” “nnet,” “gbm,” “rpart,” “GLM,” “pROC” were used for ML algorithms and “shiny” package for web application. A two tailed *P* < 0.05 was deemed statistically significant.

## Results

### Demographics Features

The clinicopathological characteristics of 1,271 PTC patients with T1-T2, non-invasive, clinically node-negative disease were summarized (Table 1). Of the 1,271 eligible patients, the average age was 42.15 ± 10.49 years (range 18–80 years). The ratio of male to female patients was 1:2.7. The mean tumor size was 9.92 mm (median = 8 mm). Eight hundred and ninety seven patients (70.6%) had papillary micro-carcinomas. Central lymph node metastases were positive in 652 (51.3%) cases.

**Table 1:** Demographic and clinicopathologic variables of the whole cohort grouped by lymph node status.

**Charteristics**	**Total (*N =* 1,271) No (%)**	**CLNM- (*N =* 619)**	**CLNM+(*N* = 652)**	***P-*value**
**Gender**				<0.001
Male	339 (26.67)	132 (38.94)	207 (61.06)	
Female	932 (73.33)	487 (52.25)	445 (47.75)	
**Age (years)**	41.38 ± 11.09	43.18 ± 11.39	39.68 ± 10.51	<0.001
≤55	1,140 (89.69)	534 (46.84)	606 (53.16)	<0.001
>55	131 (10.31)	85 (64.89)	46 (35.11)	
**Tumor size (mm)**	9.92 ± 5.69	8.53 ± 4.27	11.24 ± 6.34	<0.001
≤10 mm	897 (70.57)	491(54.74)	406 (45.26)	<0.001
10–20 mm	305 (24.00)	115 (37.70)	190 (62.30)	
>20 mm	69 (5.43)	13 (18.84)	56 (81.16)	
**Bilateral**				<0.001
No	1,071 (84.3)	544 (50.79)	527 (49.21)	
Yes	200 (15.7)	75 (37.50)	125 (62.50)	
**Tumor location**				0.127
Upper	304 (23.92)	152 (50.00)	152 (50.00)	
Middle	545 (42.88)	280 (51.38)	265 (48.62)	
Inferior	380 (29.90)	171 (45.00)	209 (55.00)	
Isthmus	42 (3.30)	16 (38.10)	26 (61.90)	
**Multifocality**				<0.001
Absence	1,002 (78.77)	514 (51.30)	488 (48.70)	
Presence	269 (21.23)	105 (39.03)	164 (60.97)	
**CLT**				0.573
No	988 (77.73)	477 (48.28)	511 (51.72)	
Yes	283 (22.27)	142 (50.18)	141 (49.82)	
**DLN status**				<0.001
Negative	1,051 (82.69)	589 (56.04)	462 (43.96)	
Positive	220 (17.31)	30 (13.64)	190 (86.36)	

*Continuous data are shown as mean ± standard deviation*.

*–, negative; +, positive; CLNM, central lymph node metastasis; CLT, chronic lymphocytic thyroiditis; DLN, Delphian lymph node*.

### Univariate and Multivariate Logistic Regression Analyses of CLNM

In univariable analysis, tumor size, gender, age, multifocality, bilateral lesions, and DLN status were all significantly associated with the occurrence of CLNM in overall population (*P* < 0.001), whereas there was no significant difference between CLNM-positive and CLNM-negative patients in terms of their tumor location or CLT status. In multivariable logistic regression analysis (Table 2), all parameters (age, gender, CLT, DLN, multifocality, bilaterality and tumor size, and location) were included. The results showed that male gender (OR 1.534, 95% CI 1.158–2.030), larger tumor size (OR 1.080, 95% CI 1.053–1.107), multifocality (OR 1.583, 95% CI 1.172–2.139), DLN metastasis (OR 6.454, 95% CI 4.246–9.651), and tumor located in inferior pole [vs. upper pole, (OR 1.507, 95% CI 1.080–2.103)] are independent positive predictors of CLNM while older age (OR 0.975, 95% CI 0.964–0.986) was a negative predictor. Variables of bilateral lesions and CLT were rejected by multivariable analysis.

**Table 2:** Univariate and multivariate logistic regression analysis of variables in predicting CLNM in whole cohort.

**Variables**	**Univariate analysis**		**Multivariate analysis**	
	**OR (95%CI)**	***P***	**OR (95%CI)**	***P***
Multifocality (+/–)	1.645 (1.250–2.165)	<0.001	1.583 (1.172–2.139)	0.003
Age	0.971 (0.961–0.981)	<0.001	0.975 (0.964–0.986)	<0.001
Gender (Male/Female)	1.716 (1.332–2.221)	<0.001	1.534 (1.158–2.030)	0.003
DLN status (+/–)	8.074 (5.392–12.092)	<0.001	6.454 (4.246–9.651)	<0.001
Tumor size (mm)	1.103 (1.077–1.130)	<0.001	1.080 (1.053–1.107)	<0.001
Tumor location		0.127		0.043
Upper	Reference		Reference	
Middle	0.946 (0.715–1.253)	0.701	1.059 (0.887–1.447)	0.719
Inferior	1.222 (0.903–1.654)	0.193	1.507 (1.080–2.103)	0.016
Isthmus	1.625 (0.838–3.151)	0.151	1.445 (0.692–3.018)	0.327
Bilateral (+/–)	1.720 (1.261–2.346)	0.001		
CLT (+/–)	0.927 (0.712–1.207)	0.573		

*DLN, Delphian lymph node; CLT, chronic lymphocytic thyroiditis; CLNM, central lymph node metastasis; –, negative; +, positive*.

### Performance of Machine Learning Algorithms

Comparisons of the performance of prediction among the six ML algorithms models in validation sets are detailed in Table 3 and Figure 1. It turned out that the XGBoost model demonstrated the highest performance of predicting CLNM, whose AUROC was 0.750, sensitivity 0.667, specificity 0.674, and accuracy 0.670 in validation sets. Accordingly, we chose the XGBoost model as the final prediction model.

**Table 3:** Predictive performance comparison of the six types of machine learning algorithms in the validation sets.

**Methods**	**AUROC**	**Sensitivity**	**Specificity**	**Accuracy**
LR	0.739	0.693	0.648	0.670
GBM	0.748	0.661	0.663	0.662
RF	0.695	0.741	0.596	0.668
DT	0.701	0.603	0.622	0.613
NNET	0.745	0.693	0.663	0.678
XGBoost	0.750	0.667	0.674	0.670

*LR, Logistic regression; GBM, Gradient boosting machine; RF, Random forest; DT, Decision tree; NNET, Neural network; XGBoost, Extreme gradient boosting*.

### Relative Importance of Variables in Machine Learning Algorithms

**Figure 2:**
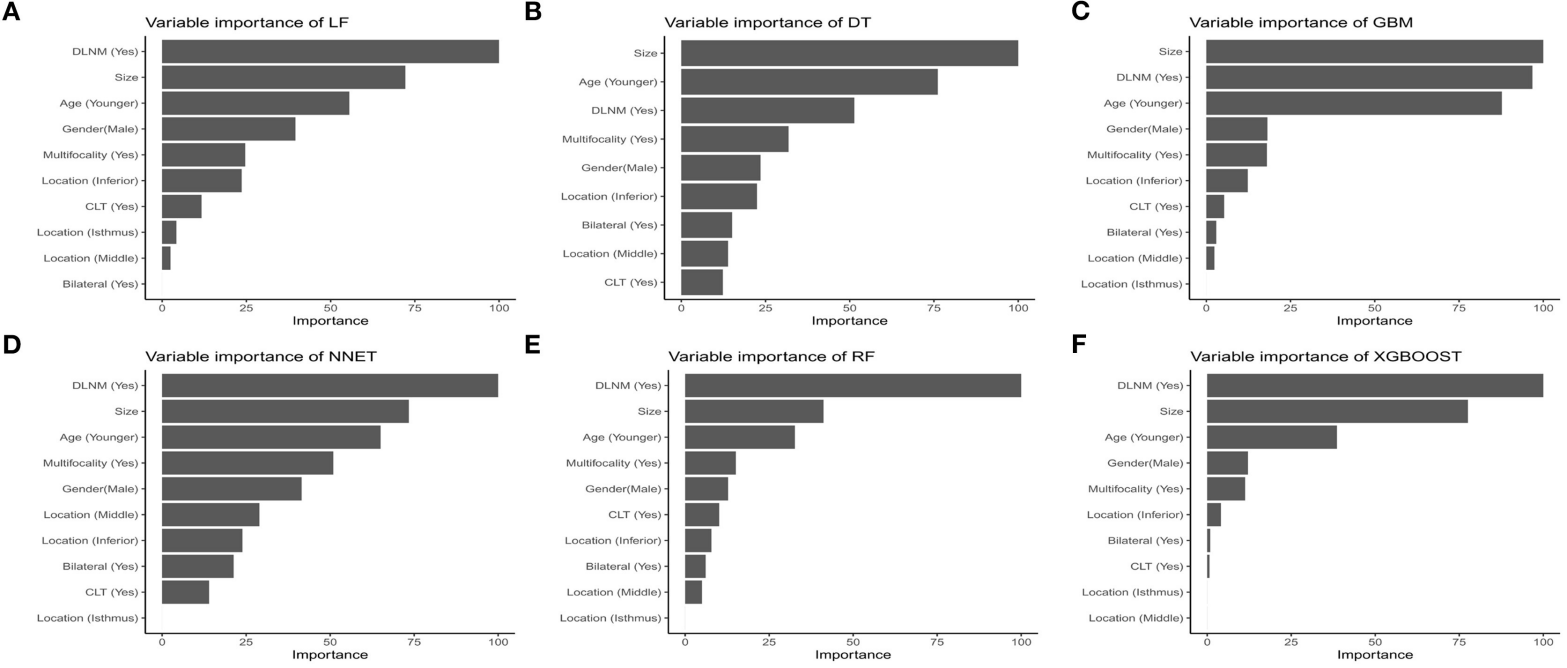
Relative importance ranking of each input variable for predition of CLNM in the machine learning algorithms. **(A)** Logistic regression. **(B)** Decision tree. **(C)** Gradient boosting machine. **(D)** Neural network. **(E)** Random forest. **(F)** Extreme gradient boosting.

The relative importance of variables in each CLNM-predicting ML algorithm is shown in Figure 2. We can see there are general trends of evidence: although slight differences are shown in the importance of variables among those ML algorithms, factors including Delphian lymph node metastasis, tumor size, age, gender, multifocality rank top five without fail. On the contrary, variables like bilateral lesions, tumor location in middle or isthmus pole and CLT make little contribution to CLNM prediction. The importance of high-ranking variables in the XGBoost model is arranged as follows in a descending order: Delphian lymph node metastasis, tumor size, age, gender, multifocality and tumor location.

### Web-Based Calculator

**Figure 3:**
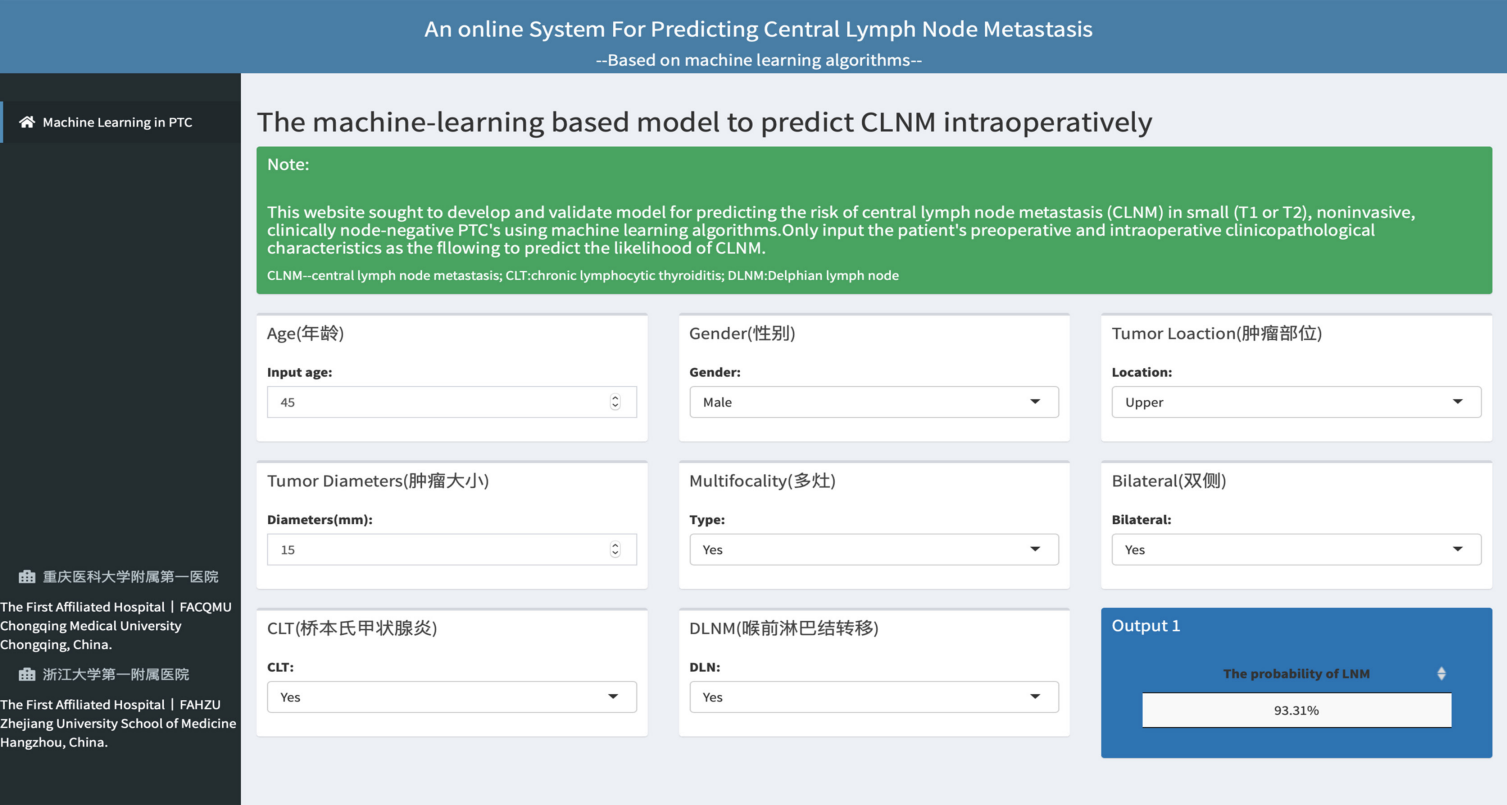
The web-based calculator for predcting central lymph node metastasis in patients with T1-T2 stage, non-invasive, and clinically node negative PTC.

An online calculator based on the best-performing model was established for clinicians to predict patients' risk of developing CLNM by simply inputing readily available preoperative and intraoperative clinicopathological variables (https://jin63.shinyapps.io/ML_CLNM/) (Figure 3).

## Discussion

In this study, we developed and validated multiple popular machine learning algorithms to predict CLNM in patients with T1-T2, non-invasive, cN0 PTC. A comparison of ML algorithms identified that the XGBoost model gave the greatest performance. To make the application of this model available, we further established an online calculator for estimating the individual probability of CLNM in this subset patients with PTC. This ML-based model may potentially guide intraoperative decision-making.

It is noteworthy that the 2015 ATA guidelines (9) asserted that “thyroidectomy without pCLND is adequate for small (T1 or T2), non-invasive, clinically node-negative PTC.” Yet, the risk of metastatic lymph nodes among this subgroup is unequal and a “one-size fits all” approach may raise concerns that in the long run it would bring potentially disastrous consequences for patients exempted from pCLND. Our data demonstrate that up to 51% of patients with T1-T2, non-invasive, cN0 PTC harbored central lymph node metastases. Such a high incidence of regional lymph node involvement is similar to other findings (18–20) and indicates that thyroid cancer is predisposed to LNM and that preoperative ultrasound currently fails to detect a massive number of patients with clinically significant lymph nodal disease (21, 22). Therefore, an accurate diagnosis of lymph node status carries much weight in helping clinicians determine the precise treatment for patients as well as informing the patients of prognoses and we advocate a selective approach to pCLND, particularly for cases with a high risk of CLNM.

Preoperative variables including larger tumors, younger age, male, multifocality, and tumor location in inferior portion are identified as the most important contributing predictors of CLNM-positive status by ML algorithms. The finding that younger age is highly predictive of CLNM in our research is similar to previous studies (23, 24). In addition, multifocal PTCs have been shown to be prone to CLNM and our results are consistent with previous reports, suggesting that multifocality is a positive predictor of CLNM (25, 26). It has been previously demonstrated by Thompson et al. (27) and Yang et al. (28) that larger tumors are significantly associated with an increased risk of nodal spread while we have found that rates of lymph node involvement surge in tumor sizes > 20 mm, compared with those in tumor sizes of 10–20 mm and < 10 mm (81.2 vs. 62.3 and 45.3%). Bilateral lesions are related with CLNM in the univariate analysis, but show insignificance in multivariate analyses after adjustment of confounders. All results have been confirmed in ML algorithms. Our study suggests that males are frequently found to be more susceptible to CLNM, which is supported by findings of previous studies (12, 29).

Nevertheless, the aforementioned factors in previous studies are mainly based on preoperative information and are still insufficient to achieve a reliable prediction. Besides, few studies have evaluated the predictive values of intraoperative factors. At our institution, lymph nodes in central compartment are classified as DLN, pretracheal and paratracheal nodes, respectively, and then routinely sent for frozen section examination separately. It was revealed in our previous study that the status of DLN based on frozen section examination was an independent predictor of CLNM and associated with poor prognostic features (14). And our findings of the present study further proves it, showing that 86.3% of DLN-positive patients have CLMN, compared with 43.9% of DLN-negative patients. The DLN status, in particular, is the strongest predictor in nearly all analytical approaches. Thus, we recommend routine intraoperative frozen section examination of DLN not only because the dissection of DLN can be performed safely without additional complications, but more importantly, it is a critical variable predicting further nodal metastases and aids in determining the extent of LN dissection. As intraoperative frozen section examination plays an essential role in immediate assessment of nodal status during an operation (30–32), it appears to be more promising in accurately predicting risks of LNM in subregion of central compartment when compared with preoperative evaluations alone.

Compared with studies attempting to predict the risk of central compartment lymph node metastases in PTC (12, 27, 28, 33, 34), our work has several strengths. First, few studies have ever focused on the subgroup of patients who suffer from clinically low-risk PTC. In fact, we found that a massive number of patients harbor clinically significant lymph node metastases which have not been detected by pre-oprative ultrasound. Furthermore, while ML approaches have shown unparalleled diagnostic performance in differentiating between benign and malignant thyroid nodules in recent reports (35, 36), there is, however, little research in the available literature on applying ML algorithms to lymph node metastases in PTC. To the best of our knowledge, this is the very first study to develop a prediction model using ML algorithms for real-time risk evaluation of CLNM with easy-to-use clinical data and fortunately, our model shows a great predictive power, which distinguishes itself from linear models adopted by previous researches. Finally, in order to make this ML-based model easy to use, we established an online application based on it, which is now available for clinicians to facilitate individualized surgical treatment by calculating the risk for each patient: (https://jin63.shinyapps.io/ML_CLNM/). For instance, if a patient is identified to have a high probability of CLNM during surgery, then pCLND may be considered despite contradiction to the current ATA guidelines.

This study, however, also has limitations. First, the nature of a retrospective study might have resulted in selection bias. Second, the ML algorithm model we established, to some extent, was confined to one single institution, which might restrict its generalizability pending further validation in real-world scenarios. Third, predictve value was not high enough because the information in our current clinical database is to a certain degree limited.

## Conclusions

We developed and validated ML algorithms for individualized prediction of CLNM in T1-T2 stage, non-invasive, and clinically node negative PTC patients by utilizing readily available preoperative variables and intraoperative frozen section examination. The ML-based prediction model can accurately identify whether patients are at high-risk of CLNM and its accompanying online risk calculator can serve as an easy-to-use tool for clinicians to make precise surgical decisions. In the future, our goal is to further integrate imaging, molecular and genetic data to improve our model performance in the realm of personalized medicine and more studies covering wider populations are also warranted for further validation.

## Data Availability Statement

The raw data supporting the conclusions of this article will be made available by the authors, without undue reservation.

## Ethics Statement

The studies involving human participants were reviewed and approved by The First Affiliated Hospital of Chongqing Medical University. The patients/participants provided their written informed consent to participate in this study. Written informed consent was obtained from the individual(s) for the publication of any potentially identifiable images or data included in this article.

## Author Contributions

JZhu, ZD, and XS conceived and designed the research. JZhu, JZhe, HR, RH, ZD, and XS prepared the manuscript. JZhu, JZhe, and DW collected and analyzed the data. JZhu, JZhe, and LL performed the statistical analysis and interpreted the results. All authors have read and approved the final manuscript.

## Conflict of Interest

The authors declare that the research was conducted in the absence of any commercial or financial relationships that could be construed as a potential conflict of interest.
