# Supplementary material for: Application of Machine Learning Algorithms to Predict Central Lymph Node Metastasis in T1-T2, Non-invasive, and Clinically Node Negative Papillary Thyroid Carcinoma
Source: Front Med (Lausanne). 2021 Mar 9;8:635771. doi: 10.3389/fmed.2021.635771 (PMC7986413; doi:10.3389/fmed.2021.635771)
Supplement: Supplementary file 1 [file Table_1.docx]

**Supplement Table. Demographic and clinicopathologics of patients in the Training and Validation sets.**

| **Charteristics** | **Training set (n=889)**  **No (%)** | **Validation set (n=382)**  **No (%)** | ***P* value** |
| --- | --- | --- | --- |
| **Gender** |  |  | 0.639 |
| Male | 241 (27.1) | 98 (25.7) |  |
| Female | 648 (72.9) | 284 (74.3) |  |
| **Age (years)** | 41.75±11.03 | 40.54±11.19 | 0.077 |
| ≤55 | 795 (89.4) | 345 (90.3) | 0.706 |
| >55 | 94 (10.6) | 37 (9.7) |  |
| **Tumor size (mm)** | 9.99±5.77 | 9.74±5.48 | 0.478 |
| ≤10 mm | 626 (70.4) | 271 (70.9) | 0.758 |
| 10 - 20 mm | 212 (23.8) | 93 (24.3) |  |
| >20 mm | 51 (5.7) | 18 (4.7) |  |
| **Bilateral** |  |  | 0.267 |
| No | 742 (83.5) | 329 (86.1) |  |
| Yes | 147 (16.5) | 53 (13.9) |  |
| **Tumor location** |  |  | 0.061 |
| Upper | 216 (24.3) | 88 (23.0) |  |
| Middle | 375 (42.2) | 170 (44.5) |  |
| Inferior | 261 (29.4) | 119 (31.2) |  |
| Isthmus | 37 (4.2) | 5 (1.3) |  |
| **Multifocality** |  |  | 0.515 |
| Absence | 696 (78.3) | 306 (80.1) |  |
| Presence | 193 (21.7) | 76 (19.9) |  |
| **CLT** |  |  | 1.000 |
| No | 691 (77.7) | 297 (77.7) |  |
| Yes | 198 (22.3) | 85 (22.3) |  |
| **DLN status** |  |  | 0.824 |
| Negative | 737 (82.9) | 314 (82.2) |  |
| Positive | 152 (17.1) | 68 (17.8) |  |
| **CLNM** |  |  | 0.032 |
| Absence | 451 (50.7) | 168 (44.0) |  |
| Presence | 438 (49.3) | 214 (56.0) |  |
| **Metastatic number of LN** |  |  | 1.000 |
| ≤5 | 812 (91.3) | 349 (91.4) |  |
| >5 | 77 (8.7) | 33 (8.6) |  |

Continuous data are shown as mean ± standard deviation

CLNM central lymph node metastasis; CLT chronic lymphocytic thyroiditis; DLN Delphian lymph node
