# Supplementary figures and images for: Application of Machine Learning Algorithms to Predict Central Lymph Node Metastasis in T1-T2, Non-invasive, and Clinically Node Negative Papillary Thyroid Carcinoma
Source: Front Med (Lausanne). 2021 Mar 9;8:635771. doi: 10.3389/fmed.2021.635771 (PMC7986413; doi:10.3389/fmed.2021.635771)

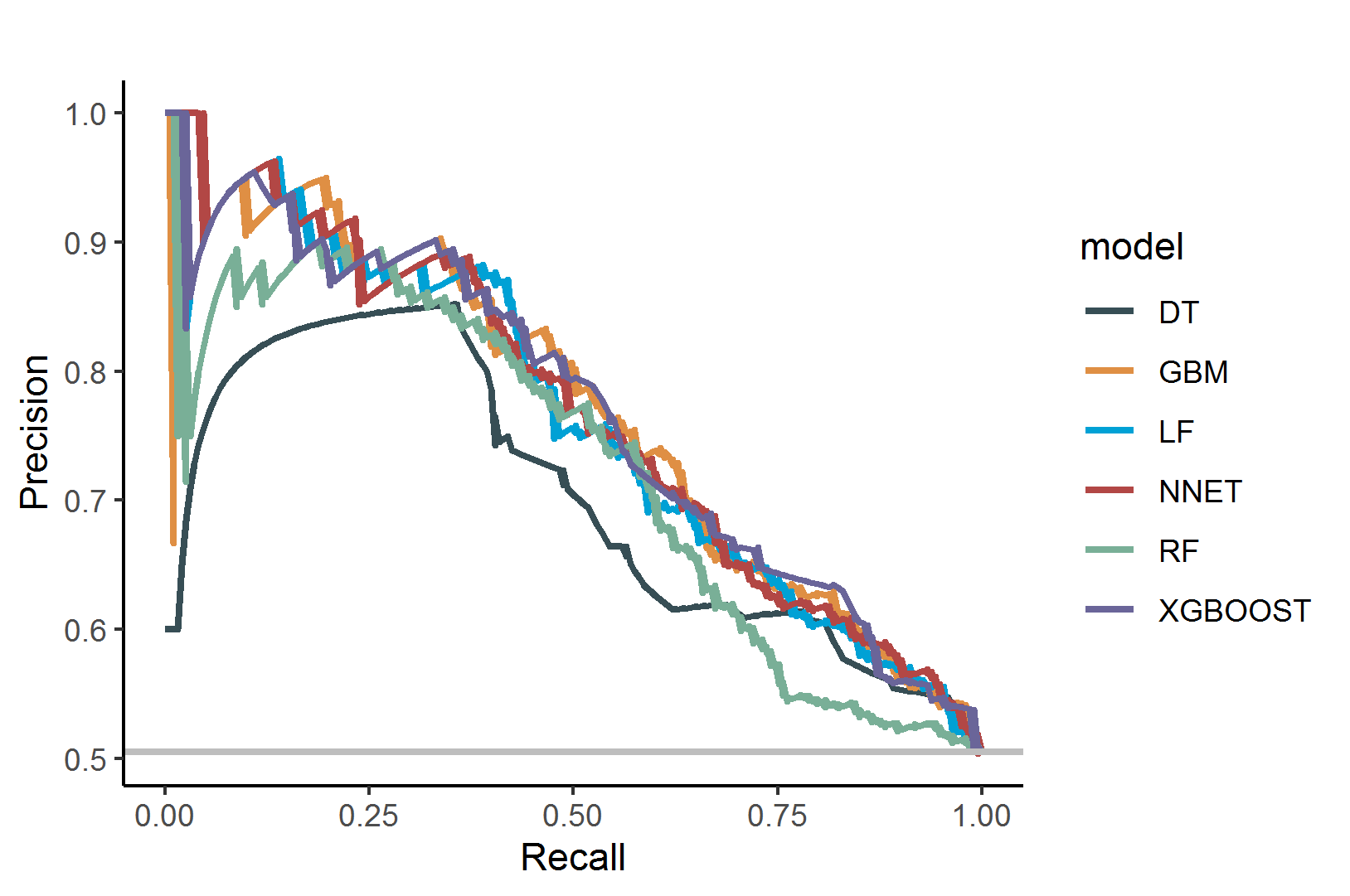

Supplement: Supplementary file 2 [file Image_1.TIFF]
